# FACTORS RELATED TO MOBILE INTERNET USE AMONG MIDDLE-AGED AND OLDER ADULTS: A MODERATING EFFECT OF DISABILITY

**DOI:** 10.1093/geroni/igac059.2174

**Published:** 2022-12-20

**Authors:** Eunjin Yang, Kyung Hee Lee

**Affiliations:** Yonsei University, Seoul, Seoul-t'ukpyolsi, Republic of Korea; Yonsei University, Seoul, Seoul-t'ukpyolsi, Republic of Korea

## Abstract

Although mobile devices are preferred as internet access devices, and the number of older adults with and without disabilities is rapidly increasing, little is known about the disability moderating effect on mobile internet use among older adults. This study examined the moderating effect of disability on older adults' mobile internet usage. This study was a secondary data analysis using 2020 Digital Divide Survey in South Korea. A total of 3,629 participants aged 55 and above were included. With the complex sample design in mind, multiple regression analysis was conducted to identify factors related to mobile internet use and test the moderating effects of disability on mobile internet use. Older adults with disabilities used mobile internet less than older adults without disabilities. However, disability status had a moderating effect on the relationships between mobile internet use and (1) operational skills of mobile devices (B=0.31, P=.004), (2) skills of internet use (B=1.46, P<.001), (3) motivation to use digital devices (B=0.46, P=.01), and (4) attitude towards new technology (B=0.50, P=.002). The results revealed that these positive relationships were stronger among older adults with disabilities than those without disabilities. Although older adults and people with disabilities are considered vulnerable populations for technology adoption, disability creates a stronger positive association between several factors and actual mobile internet use. Therefore, policymakers and practitioners should deliver tailored information and technological education for older adults with disabilities. Older adults with disabilities could be the primary beneficiaries of various mobile services.

